# Biphasic decay of the Ca transient results from increased sarcoplasmic reticulum Ca leak

**DOI:** 10.1113/JP271473

**Published:** 2016-01-06

**Authors:** Rajiv Sankaranarayanan, Yatong Li, David J. Greensmith, David A. Eisner, Luigi Venetucci

**Affiliations:** ^1^ Unit of Cardiac PhysiologyInstitute of Cardiovascular Sciences University of ManchesterManchesterUK; ^2^ Biomedical Research Centre, School of Environment & Life SciencesUniversity of SalfordSalfordUK; ^3^ Manchester Heart Centre, Manchester Royal InfirmaryCentral Manchester Foundation TrustManchesterUK

## Abstract

**Key points:**

- Ca leak from the sarcoplasmic reticulum through the ryanodine receptor (RyR) reduces the amplitude of the Ca transient and slows its rate of decay.
- In the presence of β‐adrenergic stimulation, RyR‐mediated Ca leak produces a biphasic decay of the Ca transient with a fast early phase and a slow late phase.
- Two forms of Ca leak have been studied, Ca‐sensitising (induced by caffeine) and non‐sensitising (induced by ryanodine) and both induce biphasic decay of the Ca transient.
- Only Ca‐sensitising leak can be reversed by traditional RyR inhibitors such as tetracaine.
- Ca leak can also induce Ca waves. At low levels of leak, waves occur. As leak is increased, first biphasic decay and then slowed monophasic decay is seen. The level of leak has major effects on the shape of the Ca transient.

**Abstract:**

In heart failure, a reduction in Ca transient amplitude and contractile dysfunction can by caused by Ca leak through the sarcoplasmic reticulum (SR) Ca channel (ryanodine receptor, RyR) and/or decreased activity of the SR Ca ATPase (SERCA). We have characterised the effects of two forms of Ca leak (Ca‐sensitising and non‐sensitising) on calcium cycling and compared with those of SERCA inhibition. We measured [Ca^2+^]_i_ with fluo‐3 in voltage‐clamped rat ventricular myocytes. Increasing SR leak with either caffeine (to sensitise the RyR to Ca activation) or ryanodine (non‐sensitising) had similar effects to SERCA inhibition: decreased systolic [Ca^2+^]_i_, increased diastolic [Ca^2+^]_i_ and slowed decay. However, in the presence of isoproterenol, leak produced a biphasic decay of the Ca transient in the majority of cells while SERCA inhibition produced monophasic decay. Tetracaine reversed the effects of caffeine but not of ryanodine. When caffeine (1 mmol l^−1^) was added to a cell which displayed Ca waves, the wave frequency initially increased before waves disappeared and biphasic decay developed. Eventually (at higher caffeine concentrations), the biphasic decay was replaced by slow decay. We conclude that, in the presence of adrenergic stimulation, Ca leak can produce biphasic decay; the slow phase results from the leak opposing Ca uptake by SERCA. The degree of leak determines whether decay of Ca waves, biphasic or monophasic, occurs.

## Key points

- Ca leak from the sarcoplasmic reticulum through the ryanodine receptor (RyR) reduces the amplitude of the Ca transient and slows its rate of decay.
- In the presence of β‐adrenergic stimulation, RyR‐mediated Ca leak produces a biphasic decay of the Ca transient with a fast early phase and a slow late phase.
- Two forms of Ca leak have been studied, Ca‐sensitising (induced by caffeine) and non‐sensitising (induced by ryanodine) and both induce biphasic decay of the Ca transient.
- Only Ca‐sensitising leak can be reversed by traditional RyR inhibitors such as tetracaine.
- Ca leak can also induce Ca waves. At low levels of leak, waves occur. As leak is increased, first biphasic decay and then slowed monophasic decay is seen. The level of leak has major effects on the shape of the Ca transient.

**Abbreviations**
BDM: 2,3‐butanedione monoxime
CPVT: catecholaminergic polymorphic ventricular tachycardia
ISO: isoprenaline
NCX: Na–Ca
RyR: ryanodine receptor
SERCA: sarcoplasmic reticulum Ca‐ATPase
SR: sarcoplasmic reticulum

## Introduction

Cardiac contraction is initiated by a transient increase in cytosolic Ca concentration (Ca transient) with most of this calcium released from the sarcoplasmic reticulum (SR) through the ryanodine receptor (RyR) (see Bers, 2002 and Eisner *et al*. 2013 for reviews). Normally, RyRs are shut during diastole, open to release Ca in early systole and then close to allow reuptake of Ca back into the SR by the SR Ca ATPase (SERCA). In heart failure there is a reduction in the amplitude of the Ca transient due to a depletion of SR Ca content. This can be caused by defective SERCA‐mediated Ca re‐uptake (Pieske *et al*. 1995) and/or dysfunction of RyRs that remain open in diastole, and cause Ca leak (Marx *et al*. 2001). Several studies (Bode *et al*. 2011; Andersson *et al*. 2009) have assessed the effects on Ca transient produced by impaired SERCA‐mediated Ca re‐uptake. For RyR‐mediated SR Ca leak the information available is more limited. It has been reported that leak reduces the amplitude of the Ca transient and slows its rate of decay (Negretti *et al*. 1993; Belevych *et al*. 2007; Guo *et al*. 2012). However, this has only been studied at baseline and no information is available on the effects of leak on the Ca transient in the presence of β‐adrenergic stimulation. In addition, two forms of Ca leak through the RyR have been described in heart failure: (1) Ca‐sensitising leak that is caused by an increased sensitivity of the RyR to Ca and is characterised by frequent RyR openings (Kubalova *et al*. 2005); and (2) a non‐sensitising Ca leak where channel opening is independent of Ca levels on either side of the channel and is due to locking of the channel in a sub‐conductance state (Marx *et al*. 2000). It is not clear whether there are differences in the effects on Ca transient characteristics of these two forms of leak and how they compare to the effects of SERCA dysfunction. Finally, RyR leak is also responsible for the onset of Ca waves and arrhythmias. However it is currently unclear why increased leak sometimes causes Ca waves and, at other times, results in a smaller Ca transient with a slower decay. This could be simply due to the severity of leak, with lower levels of leak causing Ca waves and more severe leak resulting in Ca waves.

A major goal of the present work was therefore to study the effects of Ca leak upon the Ca transient in the presence of adrenergic stimulation. We studied the effects of two forms of leak on Ca transient characteristics: a simple non‐sensitising leak produced by ryanodine (Rousseau *et al*. 1987) and a Ca‐sensitising leak produced by caffeine (Porta *et al*. 2011). The effects of these compounds were compared to those of the SERCA inhibitor thapsigargin, which will also decrease SR Ca content (Negretti *et al*. 1993). We also studied the effects of various levels of Ca leak on Ca waves and Ca transient. We find that in the presence of β‐adrenergic stimulation, as well as decreasing the amplitude of the Ca transient, both sensitising and non‐sensitising leak can alter its decay from monophasic to biphasic. Such biphasic decay was not produced by SERCA dysfunction. We also found that increasing leak in the presence of Ca waves induces biphasic decay and abolishes Ca waves. We conclude that biphasic decay is a typical manifestation of Ca leak and the leak necessary to induce biphasic decay is more severe than that required to generate waves of spontaneous Ca release (Venetucci *et al*. 2007). These observations provide a clear understanding of the mechanisms involved in the alterations in Ca handling caused by SR Ca leak.

## Methods

The care and use of animals were in accordance with The UK Animals (Scientific Procedures) Act, 1986 and University of Manchester Ethical Review Process. Male Wistar rats (weighing 200–250 g) were killed by stunning and cervical dislocation. Single ventricular myocytes were isolated by digestion with collagenase and protease as described previously (Eisner *et al*. 1989).

Isolated myocytes were superfused with a solution (control) consisting of (in mmol l^–1^) NaCl 135, glucose 11.1, CaCl_2_ 1, Hepes 10, MgCl_2_ 1, and KCl 4. 4‐Aminopyridine at 5 mmol l^−1^ and BaCl_2_ at 0.1 mmol l^−1^ were added to inhibit K^+^ currents, thus ensuring that tail currents on repolarization only represented the Na–Ca (NCX) currents and the solution was titrated to pH 7.4 using NaOH. Probenecid (2 mmol l^−1^) was added to reduce loss of indicator from the myocytes. Micropipettes (<5 MΩ) were filled with a solution consisting of (in mmol l^−1^): KCH_3_O_3_S 125, KCl 12, NaCl 10, Hepes 10, MgCl_2_ 5, EGTA 0.1, titrated to pH 7.2 with KOH, and a final concentration of amphotericin B of 240 g ml^−1^. Cells were voltage clamped with the perforated patch clamp technique using the discontinuous switch clamp mode (frequency 1–2 kHz and gain 0.3–0.7 nA mV^−1^) of an Axoclamp 2A voltage‐clamp amplifier (Molecular Devices, Union City, CA, USA). Cells were voltage clamped and stimulated at a range of frequencies (0.2–3 Hz) with a 40 mV, 100 ms duration pulse from a holding potential of −40 mV. All experiments were performed at room temperature.

### [Ca^2+^]_i_ measurements

The cells were incubated with the acetoxymethyl ester of Fluo‐3 (5 μm for 10 min) and allowed to de‐esterify before use. To measure changes of [Ca^2+^]_i_, at the end of each experiment the maximum fluorescence (*F*_max_) was measured by damaging the cell with the patch pipette. The dissociation constant of Fluo‐3 (*K*_d_) was taken as 400 nm (Cheng *et al*. 1993) and [Ca^2+^]_i_ calculated as described previously (Trafford *et al*. 1999).

Diastolic [Ca^2+^]_i_ was calculated by averaging [Ca^2+^]_i_ during the final 100 ms before the next stimulus. The amplitude of the Ca transient was calculated by subtracting diastolic [Ca^2+^]_i_ from peak [Ca^2+^]_i_. The rate constants of decay of the Ca transient were assessed by fitting a single or double exponential decay equation. Ca transients were analysed with custom written Excel routines (Greensmith, 2014).

In some experiments SR Ca content was estimated by releasing Ca from the SR using a mixture of 5 mmol l^−1^ caffeine and 20 mmol l^−1^ 2,3‐butanedione monoxime (BDM) and integrating the resulting NCX current (Varro *et al*. 1993; Kashimura *et al*. 2010). In the presence of SR Ca leak, diastolic Ca was significantly higher than the steady state Ca in the presence of caffeine BDM. In these cells, we integrated the NCX current to the point where [Ca^2+^]_i_ had fallen to a level comparable to that in diastole *before* application of caffeine BDM. Otherwise, the integral would have included a contribution from the elevated diastolic [Ca^2+^]_i_.

All chemicals were obtained from Sigma‐Aldrich (Poole, UK), R & D Systems (Abingdon, UK) or Fisher Scientific (Loughborough, UK). Caffeine was added as required. Ryanodine and thapsigargin were stored as 1 mmol l^−1^ stock solutions in DMSO. Thapsigargin was used to progressively inhibit SERCA (Bode *et al*. 2011).

### Statistics

Data are reported as mean ± standard error of the mean (SEM) where applicable for descriptive analysis. They were further analysed for statistical significance using *t*‐test or one‐way ANOVA. Differences were considered statistically significant at *P* < 0.05. Some experiments were analysed using the chi square test

## Results

### The effects of caffeine on the calcium transient

**Figure 1:**
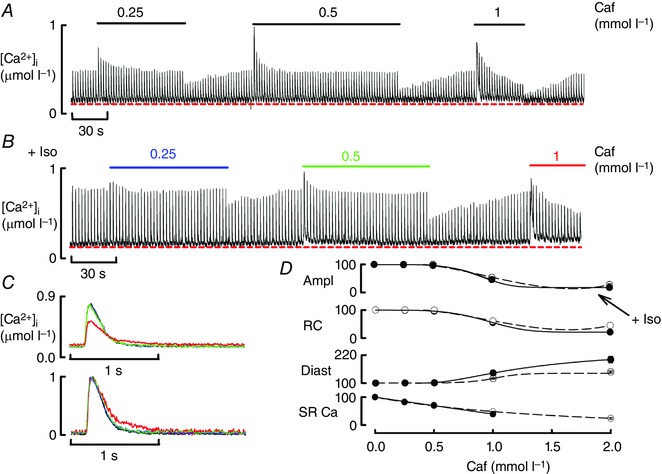
**The effects of caffeine on calcium cycling** *A*, caffeine (Caf) was applied at the concentrations indicated (mmol l^–1^) for the periods shown. *B*, effects of caffeine in the presence of isoproteronol (ISO, 1 μmol l^–1^). In both *A* and *B* the cell was stimulated at 0.5 Hz. *C*, specimen Ca transients obtained in control and in the steady state in the presence of caffeine and ISO (concentrations indicated by colours – see *B*). The top panel shows un‐normalised records and the bottom normalised. *D*, mean data showing the effects of various caffeine concentrations on (from top to bottom): Ca transient amplitude; rate constant of decay; diastolic Ca; SR Ca content. All data are normalised to values in the absence of caffeine. Points show mean and SEM for 6–8 cells (six rats). The dashed line represents baseline, and continuous line is in the presence of ISO. The amplitude, rate constant and diastolic Ca were significantly different from control (0 caffeine) at caffeine concentrations of 1 mmol l^–1^ and above. The SR Ca content was significantly decreased at all concentrations of caffeine tested.

The first set of experiments tested the effects of a sensitising leak on the Ca transient. Figure 1 *A* shows that caffeine immediately increased the amplitude of the Ca transient followed by decay to a steady level. At 0.25 and 0.5 mmol l^−1^ caffeine, this steady level had the same amplitude as the control (O'Neill & Eisner, 1990; Trafford *et al*. 2000). However, at 1 mmol l^−1^, following the initial increase, the amplitude of the Ca transient was reduced (*P* < 0.001). This was accompanied (Fig. 1 *D*) by an increase in diastolic [Ca^2+^]_i_ and a decreased rate constant of decay of the Ca transient (*P* < 0.001). Similar effects are seen in Fig. 1 *B* (from the same cell) when caffeine was applied in the presence of isoproterenol (ISO, 1 μmol l^−1^). In particular, there is a clear decrease of amplitude and slowing of decay (Fig. 1 *C*) at 1 mmol l^−1^.

### Biphasic decay of the Ca transient

**Figure 2:**
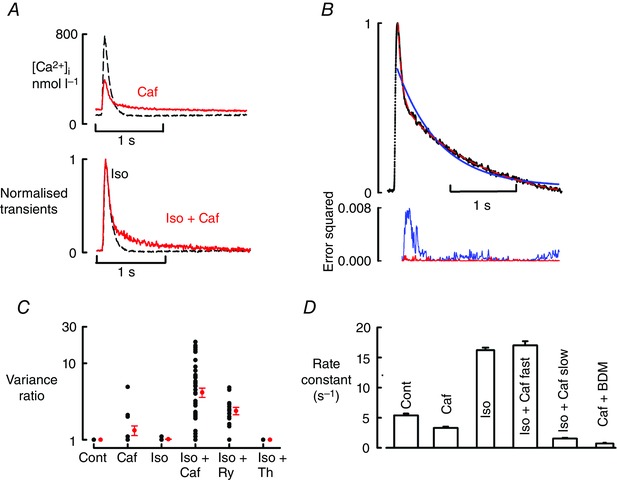
**Caffeine produces a biphasic decay of the Ca transient** *A*, upper panel, specimen Ca transients in the presence of ISO (1 μmol l^–1^). Black, dashed line obtained before and solid, red in the presence of caffeine (Caf, 1 mmol l^–1^). Lower panel, normalised traces. *B*, comparison of single (blue) and double (red) exponential fits. Top, fits to data; bottom, error squared. *C*, comparison of goodness of fit of single and double exponentials. The ordinate plots the ratio of the sum of the squares of the deviation of the data from a single exponential fit divided by that for a double exponential. The black circles show the points for individual cells and the red ones show mean and SEM. The number of cells (from left to right) is: control and caffeine, 21 cells (13 rats); Iso and Iso + Caf, 67 cells (30 rats); ryanodine (Ry), 18 cells (11 rats); thapsigargin (Th), 7 cells (fout rats). Note that in control, caffeine and ISO most of the points have values of 1.0 and therefore overlap. *D*, rate constant of decay of the Ca transient. Bars show (from left to right): control; Caf; ISO alone; fast rate constant in ISO + Caf; slow rate constant ISO + Caf; rate constant of decay of the response to adding 5 mmol l^–1^ caffeine + 20 mmol l^–1^ BDM.

A novel and striking effect observed in the presence of ISO was that the application of 1 mmol l^−1^ caffeine resulted in the appearance of a biphasic decay of the Ca transient in the majority of cells (Fig. 2 *A*). The lower (normalised) traces show that, with caffeine, the initial phase of decay was similar to that in control but that this was followed by a much slower phase. Figure 2 *B* shows that, while a single exponential (blue curve) failed to fit the data, a double (red) provided a good fit. The superiority of the double exponential fit is emphasised by the lower graph of Fig. 2 *B*. In contrast, when caffeine was added in the absence of ISO, a single exponential fit was adequate (data not shown). We have assessed the quality of the fits of the single and double exponentials by calculating the ratio of the residual variance for the single exponential fit divided by that for the double exponential fit. If both fits are equally good, this ratio will have a value of 1.0. As the double exponential fit becomes better the value will increase. As demonstrated in Fig. 2 *C*, in control solution, all the cells studied had a ratio around 1, indicating monophasic decay. In caffeine alone, only 4 of 21 cells (19%) had a ratio ≥2 showing biphasic decay. In ISO all the cells had monoexponential decays. However, in ISO plus caffeine, 47 of 67 (70 *vs*. 19% in the absence of ISO; *P* < 0.001, chi square) cells had a ratio ≥2, showing that ISO + caffeine induced biphasic decay. If leak was increased further with higher concentrations of caffeine then the fast phase of decay was lost (see Fig. 4 *E*). Mean values for the rate constants of decay are shown in Fig. 2 *D*. The rate constant of the fast phase of decay in ISO plus caffeine was as fast as the (mono‐exponential) decay constant in ISO alone (17.02 ± 0.68^^ *vs*. 16.23 ± 0.42 s^−1^; *P* = 0.32). However, the slower rate constant in ISO plus caffeine was much smaller (1.53 ± 0.13 s^−1^). We have compared the value of this slow rate constant to that seen when the SR is disabled by application of 5 mmol l^−1^ caffeine + 20 mmol l^−1^ BDM (0.73 ± 0.13 s^−1^). Under these conditions the rate constant reflects sarcolemmal Ca extrusion. Thus, from the rates of decay of the systolic and caffeine/BDM‐evoked Ca transients, SR's contribution to the fast and slow components of the decay phase can be calculated as only 50% [(1.53 – 0.73)/1.53 = 50%] to the decay of the Ca transient during the slow phase compared to 96% [(17 – 0.7)/17] during the fast phase.

### The effects of a non‐sensitising leak on systolic Ca

**Figure 3:**
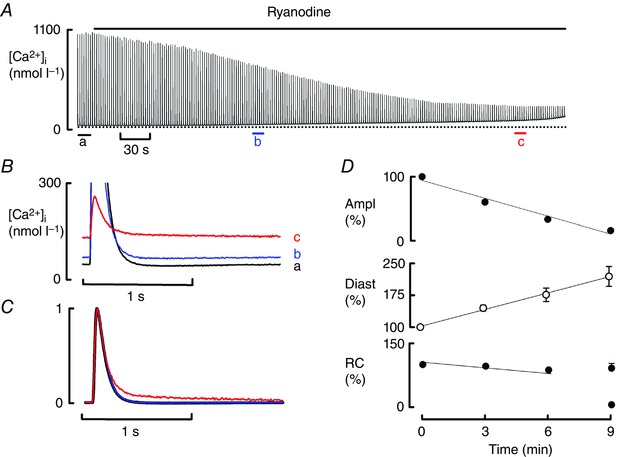
**The effects of ryanodine on Ca cycling** *A*, time course of the effects of ryanodine (1 μmol l^–1^). *B*, specimen, magnified transients to emphasise the change in diastolic [Ca^2+^]_i_. *C*, normalised transients. *D*, mean data (16 cells, 11 rats) for the time course of change of (from top to bottom): amplitude (Ampl); diastolic Ca (Diast); rate constant (RC) of decay of the Ca transient. The 9 min point shows both fast and slow rate constants. The amplitude of the Ca transient and diastolic Ca were significantly decreased at 3, 6 and 9 min. The rate of decay was only significantly deceased at 9 min (slow phase of decay).

**Figure 4:**
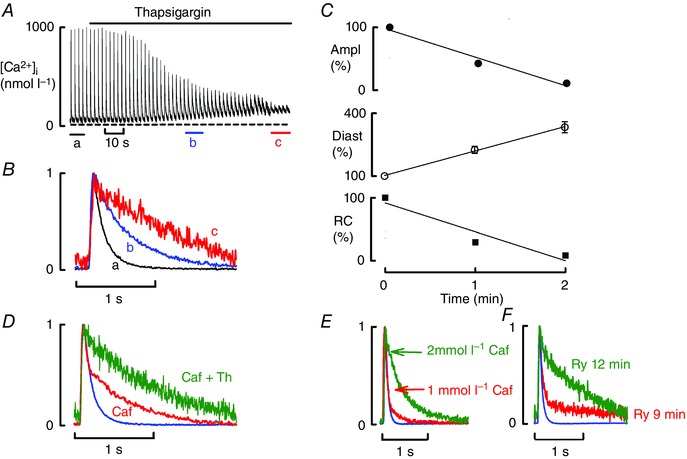
**The effects of thapsigargin on Ca handling** All data obtained in the presence of ISO (1 μmol l^–1^). *A*, time course. *B*, specimen, normalised transients from points indicated in *A*. *C*, mean data (6 cells, 4 rats) for the time course of change of (from top to bottom): amplitude (Ampl); diastolic Ca (Diast); rate constant (RC) of decay of the Ca transient. *D*, effects of adding thapsigargin in the presence of caffeine (normalised traces). *E*, comparison of the effects of 1 and 2 mmol l^–1^ caffeine (normalised traces). *F*, effects of prolonged application of ryanodine (normalised traces) showing control (ISO) and after 9 and 12 min application of ryanodine.

The next experiments investigated whether a non‐sensitising leak, produced by ryanodine, could also induce biphasic decay. Figure 3 *A* shows that 3 min of exposure to ryanodine (1 μmol l^−1^) decreased Ca transient amplitude and increased diastolic Ca but had no effect on the rate of decay of the Ca transient (Fig. 3 *Bb*). After 9 min, the reduction in Ca transient and the increased diastolic [Ca^2+^]_i_ became more marked and, in addition (similarly to caffeine), the decay of the Ca transient was slower and biphasic (Fig. 3 *B*, *C*). This biphasic decay was observed in the majority of cells (17 of 18, Fig. 2 *C*) in the presence of ISO + ryanodine and only in 1 of 7 cells in ryanodine alone (*P* < 0.001). The slow rate constant of decay (0.53 ± 0.06 s^−1^) was only slightly faster than the value of the decay when all Ca is discharged from the SR by 5 mmol l^−1^ caffeine + 20 mmol l^−1^ BDM (0.44 ± 0.1 s^−1^), again emphasising the small contribution of the SR during this phase. The mean data (Fig. 3 *D*) show that the initial decrease in Ca transient amplitude (to 60.1 ± 3.3% of control; *P* < 0.001 at 3 min) was accompanied by a gradual increase in diastolic [Ca^2+^]_i_ (to 145 ± 7% of control; *P* < 0.001) but (in contrast to caffeine) there was no significant effect (98.6 ± 1.7% of control; *P* = 0.62) on the rate constant of decay of the Ca transient at 3 or 6 min (87.4 ± 3.3%; *P* = 0.35). As was observed above with caffeine, when leak was increased beyond a certain level (here by prolonged exposure to ryanodine) the fast phase of decay was abolished and a monophasic slow decay was seen (Fig. 4 *F*).

### Comparison of the effects of increased Ca leak to those of reduced SERCA activity

We next investigated whether all manoeuvres that interfere with SR function and decrease SR Ca result in biphasic decay. Specifically, we investigated the effects of thapsigargin (an inhibitor of SERCA). As expected, thapsigargin mimicked the effects of caffeine‐induced leak in increasing diastolic [Ca^2+^]_i_ and decreasing systolic Ca transient amplitude and rate constant of decay (Fig. 4 *A–C*). However, there was no sign of a biphasic decay of the Ca transient (Fig. 4 *B*), indicating that biphasic decay is not a common feature of all forms of SR dysfunction. This conclusion is further supported by Fig. 4 *D* where the application of thapsigargin to a cell showing biphasic decay in the presence of 1 mmol l^−1^ caffeine plus ISO resulted in the abolition of the fast phase of decay and produced slow monophasic decay (see Discussion).

### Can tetracaine reverse the effects of both forms of leak?

**Figure 5:**
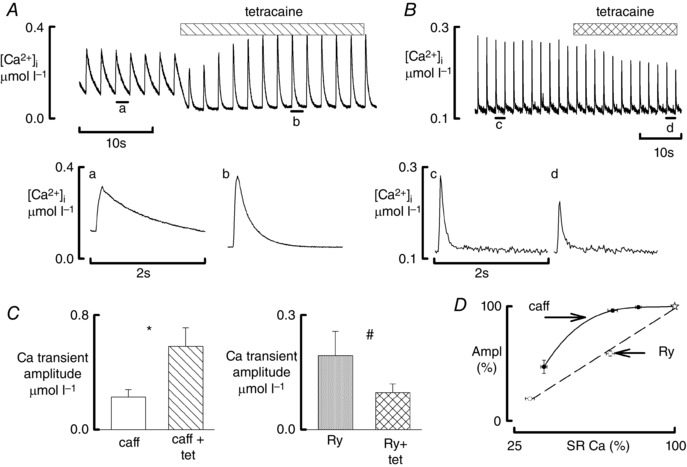
**Comparison of the effects of tetracaine in the presence of caffeine and ryanodine** All data obtained in the presence of ISO (1 μmol l^–1^). *A*, effects of tetracaine during exposure to caffeine. Top trace shows original time course. Caffeine (1 mmol l^–1^) was present throughout; tetracaine (100 μmol l^–1^) was added as shown. The lower traces show specimen records obtained at the times indicated. *B*, effects of tetracaine during exposure to ryanodine. Top trace shows original time course. Ryanodine (1 μmol l^–1^) was present throughout and tetracaine (50 μmol l^–1^) was added as shown. The lower traces show specimen records obtained at the times indicated. *C*, mean data for the effects of tetracaine on the amplitude of the Ca transient in the presence of caffeine (left: 11 cells, 4 rats) and ryanodine (right: 7 cells, 4 rats). ^#^ *P* < 0.05, *P < 0.001. *D*, dependence of the amplitude of the systolic Ca transient on SR Ca content. Data are normalised to the control values denoted by the stars. Open symbols show the effects of ryanodine (8 cells, 6 rats) and those of caffeine (10 cells, 7 rats). The right hand ryanodine points were obtained after 3 min of exposure to 1 μmol l^–1^ and the left‐hand when a markedly biphasic Ca transient was seen (8–9 min). The caffeine points show (from right to left) 0.25, 0.5 and 1 mmol l^–1^ caffeine.

Both forms of leak reduce the amplitude of the Ca transient and produce biphasic decay. Ca leak can be decreased with RyR inhibitors such as tetracaine and dantrolene (Venetucci *et al*. 2006; Maxwell *et al*. 2012). We therefore examined the effects of tetracaine on the effects of both forms of leak. We first tested the effects of 50 μmol l^−1^ tetracaine in the presence of leak induced by 1 mmol l^−1^ caffeine and detected a small but significant increase in Ca transient amplitude (data not shown). In a second set of experiments we tested the effects of 100 μmol l^−1^ tetracaine in the presence of 2 mmol l^−1^ caffeine (Fig. 5 *A*). Tetracaine increased the amplitude (Fig. 5 *A*, *C*) as well as increased the rate of decay (*P* = 0.009). In addition, in 36% of cells it transformed slow monophasic decay into biphasic decay. In contrast, in the presence of ryanodine, tetracaine *decreased* the amplitude of the Ca transient (Fig. 5 *B*, *C* right) showing that only the effects of Ca‐sensitising leak can be reversed by traditional RyR inhibitors.

### Effects of the two forms of leak on SR Ca content

Ca leak reduces the amplitude of the Ca transient by reducing SR Ca content and thence the amount of Ca released from the SR. It is therefore important to quantify the decrease in SR content produced by the two forms of leak and relate it to changes in systolic Ca transient. We measured SR Ca content in ISO and following the induction of leak either with caffeine or ryanodine. Figure 5 *D* shows the dependence of the amplitude of the Ca transient on SR Ca content for various concentrations of caffeine (solid symbols) and times of exposure to ryanodine (open symbols). In the presence of low concentrations (0.25 and 0.5 mmol l^−1^) of caffeine, initial reductions in SR content were not associated with a reduction in Ca transient amplitude. However, more severe reductions in SR content (caffeine 1 mmol l^−1^) were associated with significant reduction in Ca transient amplitude. For the ryanodine data, there were two points, corresponding to early and late effects of ryanodine. Contrary to the situation with caffeine, even a small reduction in SR Ca content produced by ryanodine decreased Ca transient amplitude. Therefore, in the presence of mild or moderate leak, a given reduction of SR Ca content induced by ryanodine caused a larger decrease of the amplitude of the systolic transient than did the same decrease produced by caffeine. However, in the presence of severe leak and biphasic decay the reductions in SR content and Ca transient produced by the two forms of leak were of a similar magnitude.

### The relationship between biphasic decay and diastolic Ca release

**Figure 6:**
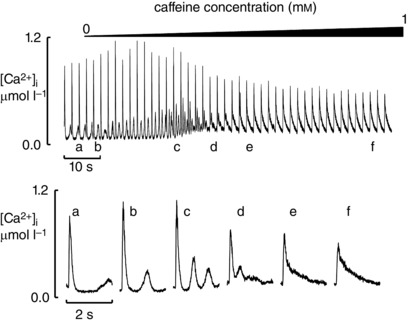
**Effects of gradual application of 1 mmol l–1 caffeine** The cell was exposed to ISO (μmol l^–1^) in an elevated Ca concentration (3 mmol l^–1^). The top trace shows the time course. Caffeine (1 mmol l^–1^) was applied with a slow solution change as shown. The lower traces show expanded records obtained at the times indicated above.

Previous work has demonstrated that increasing SR leak predisposes to the development of spontaneous Ca release and arrhythmias (Knollmann *et al*. 2006; Venetucci *et al*. 2007). We now find that leak induces biphasic decay. This raises the question of what is the difference between these two manifestations of leak? The observation that biphasic decay is observed at higher concentrations of caffeine (1 mmol l^−1^) compared to the concentration that induces Ca waves (0.25–0.5 mmol l^−1^) (Venetucci *et al*. 2007) suggests that biphasic decay is due to more severe Ca sensitisation and leak compared to the leak that induces spontaneous Ca release. To test this hypothesis we investigated the effects of a gradual increase of caffeine concentration to 1 mmol l^−1^ on cells that have already developed Ca waves (Fig. 6). This was achieved by placing the solution inflow distant from the cell. Before application of caffeine, the cell displayed Ca waves at the end of the diastolic period (a). As the concentration of caffeine within the cell gradually rose the frequency of waves increased (b–d) and waves occurred earlier during the diastolic period. At a concentration of caffeine near 1 mmol l^−1^, however, Ca waves were lost and biphasic decay occurred (e). This observation (found in all five cells studied from two rats) confirms that biphasic decay occurs when the leak is so severe that it impairs the capacity of the SR to generate waves of diastolic Ca release. In the steady state (f), the initial rapid phase of decay became smaller and most of the decay is in the slow phase (see Discussion).

## Discussion

This paper investigates the effects of RyR‐mediated Ca leak on Ca cycling. We studied the effects of two different forms of leak, Ca‐sensitising and non‐sensitising and found that, in the presence of β‐adrenergic stimulation, both can induce biphasic decay of the Ca transient. We also demonstrate that Ca‐sensitising leak can cause either Ca waves or biphasic decay and that the magnitude of the leak determines which manifestation occurs, with biphasic decay occurring in the presence of more severe levels of Ca leak.

### What causes biphasic decay of the Ca transient?

Biphasic decay is observed when sufficient leak (produced by either caffeine or ryanodine) occurs in the presence of ISO. The biphasic decay can be explained as follows. The rate of decay of the Ca transient reflects a balance between SERCA activity and RyR leak. Under control conditions, leak is so small that the decay is determined by the properties of SERCA. When leak is increased, the amount of Ca leak will be a steep function of SR Ca content (Bassani *et al*. 1995; Trafford *et al*. 1997; Zima *et al*. 2010). The initial fast phase of the decay occurs when the SR is relatively empty and the efflux of Ca is small and scarcely opposes uptake by SERCA. However, during the decay of [Ca^2+^]_i_, SR Ca content increases and therefore Ca efflux from the SR becomes larger, an effect that is made more prominent by the steep dependence of Ca leak on SR content (Zima *et al*. 2010). This will oppose Ca uptake by SERCA, thereby slowing net Ca uptake. As leak is increased further by higher concentrations of caffeine (Fig. 4 *E*) or prolonged exposure to ryanodine (Fig. 4 *F*) the initial rapid phase of decay disappears and the decay proceeds as a single, slow exponential. Presumably, under these conditions, even at low SR Ca, the leak is large enough to oppose SERCA. The fact that application of thapsigargin does not produce biphasic decay reinforces the conclusion that it results from increased leak rather than simply from impaired SR Ca re‐uptake function.

**Figure 7:**
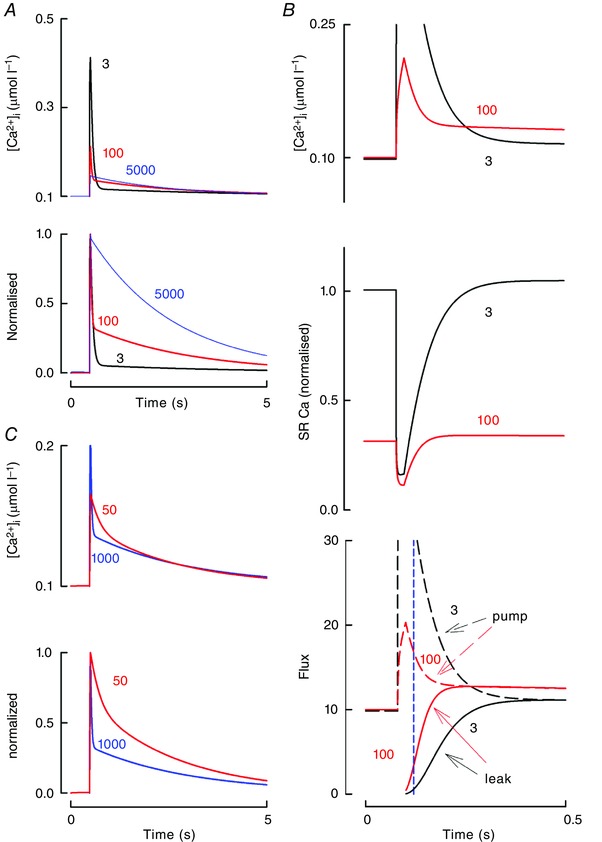
**A model for the effects of increased leak and altered SERCA activity on the systolic Ca transient** *A*, the effects of altered leak. Top, calculated transients (right hand is expanded version); bottom, normalised transients. *B*, Detailed analysis. Panels show (from top to bottom): expanded Ca transients; SR Ca; SR Ca fluxes: solid, SERCA; dashed, RyR leak. For clarity, the large Ca release during the initial phase of the Ca transient has been suppressed. Transients were calculated with the values of leak parameter (*l*) indicated. Dashed line shows *l* = 3; solid, *l* = 100; dotted, *l* = 5000. The model represents Ca efflux from the SR as *l*.{([Ca^2+^]_SR_)^3^ – ([Ca^2+^]_i_)^3^}. Ca re‐uptake into the SR is given by *k*.([Ca^2+^]_i_) where *k* has the value 1000 s^−1^. Ca efflux across the surface membrane (largely on NCX) is represented as *p*.([Ca^2+^]_i_) where *p* has a value of 50 s^−1^. Ca influx into the cell comprises two components: a constant of value 5 μmol l^−1^ s^−1^ and another, representing influx on the L‐type Ca current which, for simplicity, delivers 5 μmol l^−1^ over a 20 ms period. Cytoplasmic buffering is represented by assuming that 1% of calcium is free. The SR volume and Ca buffering are such that a given movement of Ca from cytoplasm to SR changes the free SR Ca by a factor of 10 times that of the cytoplasmic Ca. *C*, effects of altering SERCA activity. Leak (*l*) had been increased to a value of 100 in both traces. The dashed line shows SERCA activity (*k*) at 1000 and this is reduced to 50 at the continuous line.

These conclusions are supported by the model of Fig. 7 *A*. Here the magnitude of the leak is proportional to the cube of SR Ca, (SR Ca)^3^ (Trafford *et al*. 2000) multiplied by a scaling factor (*l*). The control (*l* = 3) has a rapid monophasic decay. Increasing *l* to 100 decreases SR Ca and therefore the amplitude of the transient and results in a biphasic decay (see normalised traces in lower panel). The initial rate of decay of the normalised transient is identical to the control. However, this fast phase is then followed by a slow phase of decay. This simulation therefore mimics the effects of leak produced by caffeine or ryanodine. When *l* is increased to 5000 the decay of the Ca transient becomes slow and monophasic as is seen for greater leaks (Fig. 4 *E*, *F*). The underlying events are shown in detail in Fig. 7 *B*. The top panel shows (for values of *l* of 300 and 100) an expanded version of the changes of [Ca^2+^]_i_ and the middle one those of SR Ca content. As expected, increased leak decreases SR Ca content and thence the amount of Ca released from the SR. The bottom panel of Fig. 7 *B* shows the calculated SR fluxes. The dashed lines show that (as a consequence of the decrease of the amplitude of the systolic Ca transient, the SERCA (pump) flux is decreased by increased leak. At both values of leak, the leak flux increases with time as the SR refills with Ca. Importantly, even with *l* = 100, leak is very low during the initial phase of decay of the Ca transient. For example, 0.2 s after the start of the Ca transient (blue dashed vertical line), with *l* = 100, the Ca efflux is only 0.20 of the SERCA flux. At earlier times it is an even smaller ratio and, at longer, becomes equal. In the model the leak therefore increases during the decay of the Ca transient because of an increase of SR Ca content. Two caveats need to be made. (1) The model assumes that Ca leak increases during diastole because of an increase of SR Ca. It therefore ignores the fact that recovery of the RyR from refractoriness (Belevych *et al*. 2012) may also contribute to increasing leak. This additional factor would also be expected to lead to a biphasic decay. (2) It also assumes that the dependence of SR leak on Ca content is the same for ryanodine and caffeine. We are unaware of data directly making this comparison.

### Why is biphasic decay generally seen in the presence of adrenergic stimulation?

The data and modelling show that biphasic decay requires not only leaky RyRs but also that the SR Ca content is sufficient to induce enough Ca leak through the RyRs to produce the secondary phase of decay and that SERCA activity is adequate to have the initial fast rate of decay. Adrenergic stimulation will stimulate SERCA and increase SR Ca content by promoting phosphorylation of phospholamban. Further support for this explanation is provided by the observation that biphasic decay disappears following inhibition of SERCA with thapsigargin (Fig. 5 *D*). This is modelled in Fig. 7 *C*. Here, both traces show the effect of increased leak. As shown by the continuous line, SERCA activity has been decreased, resulting in the virtual abolition of the biphasic decay. Finally, the effect of leak on the decay of the Ca transient emphasises that, at least in the presence of significant leak, the rate of decay is not governed simply by SERCA but, rather, by the combined effects of SERCA and leak.

One issue that needs to be considered is that, as well as affecting the RyR, caffeine is a phosphodiesterase inhibitor. It might therefore be expected to increase both Ca influx through the L‐type current and SERCA activity and either could contribute to biphasic decay. It is therefore important to note that caffeine concentrations up to 1 mm had no effect on the L‐type current in the presence of ISO. Furthermore, ryanodine, which is not known to affect phosphodiesterases, also produces biphasic decay.

### Relevance of biphasic decay

It is important to consider why the increase of Ca leak observed in heart failure has not (to the best of our knowledge) been associated with a biphasic decay. One explanation is that many forms of heart failure are characterised by reduced SERCA activity (Gwathmey *et al*. 1987; Nagai *et al*. 1989; Mercadier *et al*. 1990; Hasenfuss *et al*. 1994) and this (Fig. 5 *D*) would be expected to make biphasic decay less likely. However, note that, in some models of heart failure, reduction of the Ca transient is exclusively due to severe RyR‐mediated Ca leak (Belevych *et al*. 2007). It would be important to study whether the maintenance of SERCA activity in this model means that increased leak can produce biphasic decay. Another clinical condition associated with RyR‐mediated Ca leak is catecholaminergic polymorphic ventricular tachycardia (CPVT). This is a genetic arrhythmia syndrome characterised by the onset of life‐threatening ventricular arrhythmias during adrenergic stimulation of the heart that typically occurs during exercise and emotional stress. It is caused by mutations of RyR or its accessory protein calsequestrin (Priori *et al*. 2002). The mutations increase RyR opening, cause Ca leak and predispose to the development of Ca waves, delayed after‐depolarisations and arrhythmias. The mutations have similar effects to those of low concentrations of caffeine (Nam *et al*. 2005) and, consistent with this, CPVT mutations can reduce SR Ca content without a significant change in Ca transient amplitude (Kashimura *et al*. 2010). Interestingly a recent paper (Liu *et al*. 2013) studying the effects of a calsequestrin mutation (R33Q) associated with CPVT detected alterations of the Ca transient that are consistent with severe Ca leak. These included a reduction in Ca transient amplitude and a slowing of the rate of decay. Following adrenergic stimulation some cells (their data supplement Fig II) clearly developed biphasic decay, again an expected consequence of leak.

Another important issue is whether biphasic decay could have any pro‐arrhythmic effect. Figure 6 showed that biphasic decay removed Ca waves. The expected abolition of delayed after‐depolarisations would suggest that biphasic decay will not have pro‐arrhythmic effects. The only potential pro‐arrhythmic effect could be related to increased NCX inward current associated with the slow decay that could cause action potential prolongation, but this would depend on the interaction with other membrane currents.

### Are there differences between sensitising and non‐sensitising Ca leak?

Although these two forms of leak produce qualitatively similar effects on the Ca transient, there are some important differences. The most important relates to the alterations in channel gating produced by these two agents. As mentioned above, ryanodine locks a subset of channels in a sub‐conducting state (Rousseau *et al*. 1987). The remainder of the channels (which are not affected by ryanodine) continue to gate normally. The magnitude of the leak is determined by the number of channels that are in this sub‐conducting state and by the driving force for Ca flux through the channel, which, in turn, depends on SR Ca content. In contrast, caffeine affects the gating of all the RyRs in the cell and increases their sensitivity to Ca (Porta *et al*. 2011). This results in the potentiation of RyR opening and Ca released by the SR (increased fractional release) for a given SR Ca content. On the basis of these considerations it is easy to explain the different relationship between SR Ca content and Ca transient amplitude observed in the two forms of leak. The amplitude of the Ca transient is a steep function of SR Ca content and therefore a small reduction in SR Ca content produced, for example, by early ryanodine application results in a marked reduction of the amplitude of the Ca transient. However, in the presence of caffeine the sensitisation of the RyR and the potentiation of release compensates for the depletion in SR until the point where despite full opening of the RyR (100% fractional release) there is not enough Ca in the SR to maintain the amplitude of the Ca transient (Negretti *et al*. 1993). This explains why in the presence of ryanodine small decreases in SR Ca content produce significant reduction in Ca transient amplitude, while in the presence of caffeine a much greater reduction in SR content is needed to reduce the Ca transient. However, when leak becomes very severe and produces biphasic decay, the degree of SR Ca depletion and reduction in Ca transient produced by the two forms of leak becomes similar. In previous work we have stressed that potentiation of the RyR (with low concentrations of caffeine) results in no change in the amplitude of the Ca transient (Trafford *et al*. 2000). This results from the need for the cell to remain in Ca flux balance. The fact that higher concentrations of caffeine and therefore levels of leak decrease the amplitude of the Ca transient means that Ca flux balance must be achieved by a combination of increased diastolic [Ca^2+^]_i_ and slowed rate of decay of the Ca transient compensating for the decrease of amplitude (Eisner *et al*. 2013). Likewise, the increase of Ca transient amplitude produced by tetracaine reducing leak (Fig. 5) is accompanied by a decrease of diastolic [Ca^2+^]_i_, thereby tending to restore Ca efflux and flux balance.

Another important difference between these two forms of leak is their response to tetracaine. Our data clearly show that only the effects of Ca‐sensitising leak can be reversed by tetracaine. Presumably the ryanodine‐modified RyR is insensitive to tetracaine. Over the last few years, several RyR inhibitors have been developed to treat heart failure and CPVT. The effectiveness of all these compounds will depend on what form of leak is present in heart failure and whether these compounds affect RyR Ca sensitivity. This is certainly an issue that needs to be clarified to understand whether these agents can be effective in the treatment of heart failure.

### The occurrence of biphasic decay or Ca waves depends on the magnitude of sensitising leak

In previous work we demonstrated that sensitising leak induced by low concentrations caffeine induces Ca waves (Venetucci *et al*. 2007). We now show that caffeine can also induce biphasic decay and that the concentrations of caffeine and the levels of leak necessary for the induction of biphasic decay are higher than those needed to induce Ca waves. In simple terms, what determines the occurrence of Ca waves or biphasic decay is the degree of Ca leak. We can easily explain this observation if we consider that caffeine and other Ca‐sensitising manoeuvres, in addition to inducing Ca leak, shorten Ca release restitution (Ramay *et al*. 2011; Belevych *et al*. 2012). At intermediate levels of leak and sensitisation full restitution of Ca release will occur in mid‐diastole and this will facilitate the occurrence of Ca waves. At higher levels of Ca sensitisation restitution of Ca release will occur much earlier during the decay of the Ca transient and this will cause biphasic decay. This interpretation is supported by the experiments performed in Fig. 6 that clearly show that, with increasing levels of caffeine and sensitisation, Ca waves occur earlier in the diastolic period until they merge with the Ca transient and cause biphasic decay. As leak is increased further the fast phase of decay disappears and a monoexponential, slow decay results.

### Limitations

It is important to consider some limitations of the present study. (1) It was performed on rats which have higher levels of SERCA‐mediated Ca removal compared to larger mammals where NCX plays a more prominent role. Biphasic decay requires high levels of SERCA activity to mediate the first phase of the decay and load the SR. This raises the question of whether biphasic decay would occur in larger mammals. We would expect qualitatively similar effects with a less prominent secondary phase. From the data available, it is difficult to have a certain answer and therefore future work should investigate this in other species. (2) Another limitation relates to the voltage step used to stimulate the cells. Cells were stimulated using a 100 ms voltage step from −40 to 0 mV. This is different from the shape of the action potential in rat and raises the question of whether in the presence of the normal action potential biphasic decay would still be observed. The voltage step is longer than the action potential and the holding potential more depolarised compared to the normal resting potential. These differences would be expected to result in a rat action potential favouring NCX activity and promoting SR unloading, and this reduced load would reduce Ca leak. However, it is important to remember that recent work has suggested that Ca leak is more prominent when cells are stimulated from −80 mV because the increased Na current increases Na levels in the dyadic space (Sikkel *et al*. 2013). This would favour biphasic decay. Our voltage step is shorter than the action potential of larger mammals and therefore one would expect more SR loading in the presence of a mammalian action potential and this should again favour leak and biphasic decay This certainly needs to be addressed. (3) Finally, our experiments were performed a room temperature. SERCA and RyR activity are modulated by temperature and this raises the question of whether biphasic decay could be seen at 37^ ^°C. Our work has demonstrated that biphasic decay occurs when severe SR leak is associated with maximised SERCA activity. Changing temperature may modify the experimental conditions needed to observe biphasic decay but should not stop it occurring.

## Additional information

### Conflict of interest

The authors declare that they have no conflicts of interest.

### Author contributions

LV, DAE, RS and DG designed the experiments and analysed the data; RS and YL performed the experiments; RS, LV and DAE wrote the paper; all authors revised and approved the final version of the manuscript.

### Funding

The work in the paper was supported by grants from the British Heart Foundation.
